# Impact of Long-Term Exposure to the Tyrosine Kinase Inhibitor Imatinib on the Skeleton of Growing Rats

**DOI:** 10.1371/journal.pone.0131192

**Published:** 2015-06-24

**Authors:** Josephine T. Tauer, Lorenz C. Hofbauer, Roland Jung, Sebastian Gerdes, Ingmar Glauche, Reinhold G. Erben, Meinolf Suttorp

**Affiliations:** 1 Department of Pediatrics, University Hospital Carl Gustav Carus, TU Dresden, Dresden, Germany; 2 Department of Internal Medicine III, University Hospital Carl Gustav Carus, TU Dresden, Dresden, Germany; 3 Experimental Center of the Medical Faculty Carl Gustav Carus, TU Dresden, Dresden, Germany; 4 Institute for Medical Informatics and Biometry, Medical Faculty Carl Gustav Carus, TU Dresden, Dresden, Germany; 5 Department of Biomedical Sciences, University of Veterinary Medicine, Vienna, Austria; B.C. Cancer Agency, CANADA

## Abstract

The tyrosine kinase (TK) inhibitor imatinib provides a highly effective therapy for chronic myeloid leukemia (CML) via inhibition of the oncogenic TK BCR-ABL1. However, off-target TKs like platelet-derived growth factor receptors (PDGF-R) and colony-stimulating factor-1 receptor (c-fms), involved in bone remodeling, are also inhibited. Thus, pediatric patients with CML on imatinib exhibit altered bone metabolism, leading to linear growth failure. As TKI treatment might be necessary for a lifetime, long-term effects exerted on bone in children are of major concern. Therefore, we studied the skeletal long-term effects of continuous and intermittent imatinib exposure in a juvenile rat model.

Four-weeks-old male Wistar rats were chronically exposed to imatinib via drinking water over a period of 10 weeks. Animals were exposed to a standard and high imatinib dosage continuously and to the high imatinib dose intermittently. Bone mass and strength were assessed using pQCT, micro-computed tomography (μCT), and biomechanical testing at the prepubertal, pubertal, and postpubertal age. Bone length and vertebral height as well as biochemical markers of bone turnover were analyzed.

Femoral and tibial bone length were dose-dependently reduced by up to 24% (p<0.0001), femoral and tibial trabecular bone mass density (BMD) were reduced by up to 25% (p<0.01), and femoral breaking strength was lowered by up to 20% (p<0.05). Intermittent exposure mitigated these skeletal effects. Long-term exposure resulted in reduced vertebral height by 15% and lower trabecular BMD by 5%. Skeletal changes were associated with suppressed serum osteocalcin (p<0.01) and non-significantly elevated serum CTX-I and PINP levels.

In conclusion, imatinib mainly impaired longitudinal growth of long bones rather than the vertebrae of growing rats. Interestingly, intermittent imatinib exposure has less skeletal side effects, which may be beneficial in pediatric patients taking imatinib.

## Introduction

Chronic myeloid leukemia (CML) is characterized by a balanced reciprocal chromosomal translocation involving the *ABL1* gene on chromosome 9 and the *BCR* gene on chromosome 22, building the *BCR-ABL1* fusion gene, which encodes for the constitutively activated tyrosine kinase (TK) BCR-ABL1. TK inhibitors (TKIs) like imatinib have been developed to bind to the ABL1 subunit of BCR-ABL1 and thereby to suppress phosphorylation of proteins involved in signaling cascades necessary for leukemic cell growth. Imatinib treatment has become an established treatment of CML and has been approved for adult CML in 2001 and also as frontline therapy for pediatric CML in 2003 [1–3]. Development of resistance to imatinib due to point mutations in the ABL1 domain may occur, and has promoted the development of more potent 2^nd^ and 3^rd^ generation TKIs. Up to know, none of these TKIs seem to eliminate the malignant leukemic cell clone completely, resulting in a life-long treatment in the majority of the patients with CML to sustain the remission. For pediatric patients this means a treatment period comprising decades starting in childhood, during puberty, and adolescents. This raises the question of long-term side effects of TKI therapy.

*In vitro* kinase inhibition assays [4] revealed that beside BCR-ABL1 imatinib also exerts off-target effects on further TKs such as stem cell growth factor receptor (c-KIT), platelet-derived growth factor receptors (PDGF-R), and colony-stimulating factor-1 receptor (c-fms) that are involved in bone metabolism. Stimulation of c-fms promotes the differentiation of monocytic progenitors to bone-resorbing osteoclasts. In addition, signaling cascades involving PDGF-R and c-ABL1 regulate differentiation of bone-forming osteoblasts. In adult patients with CML the imbalance between bone formation and resorption results in disturbed biochemical serum markers of calcium and phosphate homeostasis, increased bone mineralization, and increased trabecular bone volume [5,6]. Pediatric patients with CML suffer linear growth failure from imatinib treatment, being more pronounced in prepubertally diagnosed patients compared to pubertal patients [7–10].

Taken together, impaired bone remodeling is observed as side effect of TKI therapy. However, so far, skeletal side effects of continuous imatinib treatment have been investigated *in vivo* in adult rats only [11,12]. Thus, the detailed action of imatinib on the growing bone is insufficiently examined.

To elucidate the side effects of imatinib treatment on i) bone growth and ii) at defined developmental stages, we established a juvenile rat model of chronic imatinib exposure and characterized the subsequent changes in bone metabolism. Furthermore, based on an effective anti-leukemic treatment strategy in adult patients with CML [13,14], we tested the hypothesis whether intermittent imatinib treatment can minimize skeletal side effects. Here, we show that continuous long-term imatinib exposure results in juvenile rats in diminished bone length, bone mineral density, and bone strength in a dose-dependent fashion, whereas intermittent imatinib exposure minimizes these untoward bony effects.

## Materials and Methods

### Animals and experimental design

Juvenile male Wistar rats (Elevage Janvier, Le Genest St. Isle, France) were chronically exposed to varying concentrations of imatinib via the drinking water. During the entire exposure time of 10 weeks—starting at the age of 4 weeks until 14 weeks—the critical developmental stages from end of weaning until young adolescence are covered. A high (2 mM in drinking water) and a standard dosage (1 mM) of imatinib were administered continuously during the experiment while untreated controls received water only. Another cohort was exposed to the high dosage intermittently (3 consecutive days “on” and 4 days “off”). Due to lab space limitations, two consecutive experiments under identical conditions were performed. Based on statistical power calculation a total number of 26 animals per cohort were investigated. Juvenile rats were kept under standardized specific pathogen-free conditions at 21°C and 12 h/day lightning (07:00–19:00 hrs) with free access to food and water. Behavior and weight gain of all animals were monitored 3 times weekly. All experiments were carried out in accordance with the Institutional Animal Care and Use Guidelines and were approved by the Agency for the Inspection of Veterinary and Food Materials of the Government of Saxony (permit number 24–9168.11-1/2009-16).

### Analysis of drug blood levels, long bone length, and vertebral height

At prepubertal stage (age 6 weeks; after 2 weeks of exposure), at pubertal stage (age 8 weeks; after 4 weeks of exposure), and at postpubertal stage (age 14 weeks; after 10 weeks of exposure), 8–10 animals from each cohort were humanely killed. Blood serum was collected and long bones and lumbar vertebrae L1-L4 were isolated. Drug serum levels were measured using HPLC as described elsewhere [15]. Length of both femora and tibiae were measured using a digital caliper with a resolution of 0.01 mm (Merox, Vienna, Austria). Height of the vertebral body L2 was defined as distance between the two intervertebral discs measured using the scout-view mode of the peripheral quantitative computer tomography (pQCT) machine.

### Analysis of bone mineral density (BMD) by pQCT

BMD of the right femur, tibia, and vertebra L2 of all rats was investigated by pQCT using a XCT Research SA+ machine (Stratec Medizintechnik, Pforzheim, Germany). Measurements were undertaken on specimens stored in 70% ethanol with a collimator opening of 0.2 mm, and a voxel size of 70 μm. Initially, a coronal computed radiograph (scout view) was performed. For long bones the reference lines were set at the distal growth plate of the femora and at the proximal growth plate of the tibiae, respectively. Two slices for BMD calculation were defined: i) one slice was positioned in the secondary spongiosa located proximal to the distal growth plate of the femora and in the secondary spongiosa located distal to the proximal growth plate of the tibiae for assessment of trabecular BMD, and ii) the other slice was positioned in the mid-diaphysis of femora and tibiae for cortical BMD measurement of the cross-sectional area (CSA). For determination of trabecular BMD, the distance to the reference line was defined according to bone growth depending on animal age to ensure analysis of the same region at every developmental stage: femoral trabecular BMD was analyzed in prepubertal bone at 3.4 mm, in pubertal bone at 3.9 mm, and in postpubertal bone at 4.0 mm proximal to the distal growth plate while tibial trabecular BMD was analyzed in prepubertal bone at 2.6 mm, and in pubertal and postpubertal bone, respectively, at 2.7 mm distal to the proximal growth plate.

For BMD analysis of the vertebral body L2, the mean of three slices was calculated. The first slice was positioned in the middle of the vertebral body and another two slices each 1 mm above and below. Thresholds of 280 mg/cm³ and 710 mg/cm³ were defined for trabecular and cortical BMD calculation. Image acquisition, processing, and calculation of the results were performed using the software package provided by the manufacturer (XCT 6.00, Stratec, Pforzheim, Germany).

### Three-point bending strength of the femora

Following pQCT measurements, the right femora of 3–4 rats per cohort were subjected to mechanical testing by a three-point bending test [16], using a Z020 material testing machine (Zwick/Roell, Ulm, Germany) and the corresponding analysis software TestXpert II (Zwick/Roell, Ulm, Germany) as published elsewhere [17]. Briefly, three-point bending strength was analyzed using a 1 kN force detector with a force resolution of 0.01 N. Each bone was compressed with a constant speed of 2 mm/min until fracture. Breaking force was defined as bending load at fracture F_max_ [N].

### Analysis of trabecular structure of long bones

Three dimensional trabecular microarchitecture of the right femora of 3–4 rats per cohort was investigated with a μCT machine (μCT35, SCANCO Medical, Brüttisellen, Switzerland). Bones were scanned at 70 kVp/114 μA with an isometric voxel resolution of 12 μm. A trabecular region of interest was defined manually in the distal femora in a distance of 500 μm to the growth plate and adapted to bone growth to ensure analysis of the same region at every developmental stage. 196 slices located within the secondary spongiosa in prepubertal femora, 226 slices in pubertal femora, and 256 slices in postpubertal femora, respectively, were analyzed. Digital segmentation of the bone from air/soft tissues was performed by adaptive (median-C) thresholding. For trabecular bone regions, bone volume fraction (bone volume/ total volume; BV/TV), trabecular thickness (Tb.Th), trabecular number (Tb.N), and trabecular connectivity (Tb.C) were calculated. To separate mineralized matrix from surrounding tissue a BMD threshold of 211 mg/cm³ was defined [18].

### Analysis of bone metabolic serum parameters by ELISA

Cardiac blood samples were collected and spun at 10,000 x g for 10 minutes to collect serum and stored immediately at −80°C until analysis. Serum tartrate-resistant acid phosphatase (TRAP), C-terminal collagen cross-links (CTX-1), osteocalcin, procollagen type I (PINP), and parathyroid hormone (PTH) were measured by ELISA, as described by the manufacturer (Immunodiagnostic Systems, Frankfurt, Germany). Inter- and intraassay variations ranged from 3%- 10%, respectively.

### Statistical analysis

ANOVA test of the effects at the three defined time points explicitly accounting for the individual experiments revealed no systemic batch effect of the two experiments. Thus, for statistical analysis, we pooled the data of both experiments to achieve a better estimate of the true population variability. Effects of imatinib treatment on bone metabolism were evaluated in comparison to age-related controls in two steps.

First, imatinib effects on growth were estimated by linear regression during the entire 10-week exposure time. For statistical analysis of the body weight by linear regression, measurements at start and week 2, 4, and 10 of exposure, respectively, were treated as equidistant to ensure linearity of the regression. Therefore, reported ‘growth rates’ correspond to mean growth per interval (start to week 2, week 2 to week 4, week 4 to week 10) rather as to growth rate per week.

Second, to disclose imatinib effects according to developmental stages, ANOVA was used to estimate effects at the defined time points pre-, pubertal, and postpubertal. Paragraphs of the results sections are structured by first describing observed skeletal effects by imatinib on bone growth over 10-weeks of exposure and afterwards separately at the three mentioned developmental stages. In the text, skeletal effects of imatinib exposure are given in % in relation to age-related controls. Unless otherwise specified, results presented in tables and figures are showing mean ± 95% CI.

For statistical analysis the free software “R i386 3.1.1” was used and p values <0.05 were defined as statistically significant. Graphical illustrations were prepared using GraphPad Prism 5.0 software (GRAPHPAD Software, San Diego, USA).

## Results

### Animal development, body weight, and fluid intake

At start of TKI exposure rat’s mean body weight was 68.5 ± 9.2 g (S1 Fig). Monitoring of body weight of untreated controls revealed a mean ‘weight gain rate’ of 124.92 ± 1.79 g/interval (Table 1). During entire exposure time, body weight of TKI treated animals revealed mean ‘weight gain rate’ of 122.45 ± 1.85 g/interval in rats chronically exposed to 1 mM imatinib and 117.23 ± 1.89 g/interval intermittently exposed to 2 mM imatinib. However, body weight of rats exposed continuously to 2 mM imatinib revealed a significantly decreased mean ‘weight gain rate’ of 103.87 ± 1.82 g/interval (p<0.001; Table 1). However, no alterations of exposed animals, with regard to their overall development and behavior (e.g. movement in the cage, texture of the hair, clear eyes, stool consistency), were observed in comparison to untreated controls.

**Table 1:** Body weight during long-term imatinib exposure.

	**Exposure (weeks)**				**‘weight gain rate’ per interval** ^¥^
	**Start**	**2**	**4**	**10**	
**Control**	67.9±9.2 g	200.9±12.4 g	303.2±17.4 g	449.1±27.4 g	124.9±1.8 g
**Imatinib 1 mM**	68.6±10.3 g	194.1±9.3 g	300.3±10.7 g	431.2±26.2 g	122.5±1.9 g
**Imatinib 2 mM**	68.9±9.9 g	169.6±13.6 g	264.2±14.9 g	388.7±24.4 g	103.9±1.8 g***
**Imatinib 2 mM on/off**	68.6±7.9 g	178.8±13.8 g	274.7±10.3 g	440.1±34.6 g	117.2±1.9 g

Data represents mean ± standard deviation.

^¥^ ‘Weight gain rate’ was calculated by linear regression analysis estimating body weight at start and week 2, 4, and 10 of exposure as equidistant to ensure linearity of the regression. Therefore, reported ‘weight gain rate’ corresponds to mean weight gain per interval (start to week 2, week 2 to week 4, week 4 to week 10).

*** p<0.001 versus age-related controls.

**Fig 1:**
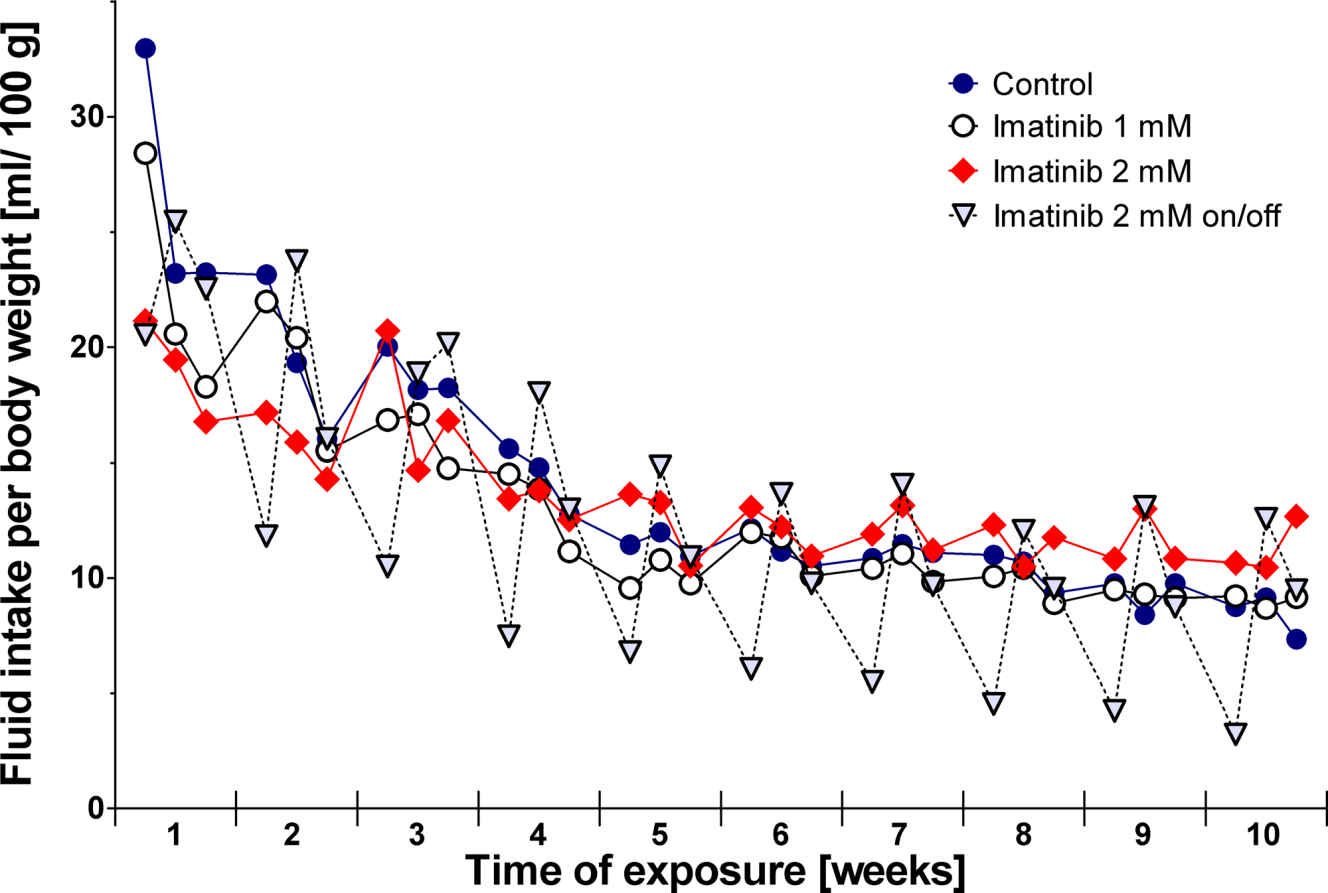
Fluid intake of the rats during long-term imatinib exposure. Fluid intake was measured 3 times weekly (Monday, Wednesday, Friday). Data represents mean ± 95% CI.

Monitoring of drinking patterns revealed no differences in rats exposed to imatinib continuously compared to controls (Fig 1). During the 10-week period of exposure, controls consumed a mean volume of 14.0 ± 5.7 mL/100 g body weight each day, rats exposed to 1 mM imatinib drank 13.0 ± 4.7 mL/100 g body weight, and rats exposed to 2 mM imatinib drank 13.7 ± 2.9 mL/100 g body weight. However, in rats exposed intermittently to 2 mM imatinib an altered drinking pattern was observed starting in the third week of exposure. At days exposed to imatinib the animals consumed less water than compared to the days without imatinib exposure: during exposure daily fluid intake was 8.0 ± 4.8 mL/100 g body weight while this volume was increased to 15.1 ± 5.2 mL/100 g body weight at days off imatinib exposure (Fig 1). However, the overall mean weight gain and overall development of this cohort during total exposure time did not differ from controls.

### Imatinib drug serum levels

The mean ingested imatinib doses (mg/kg/d) were calculated based on the drinking volume and body weight of the animals. Rates exposed to 1 mM imatinib, 2 mM imatinib, and 2 mM imatinib intermittently, respectively, ingested 64.4 ± 23.6 mg/kg/d, 137 ± 29.1 mg/kg/d, and 80.2 ± 47.5 mg/kg/d, respectively, during the 10-week exposure period. Mean serum levels of imatinib were 1678 ± 481 ng/mL after 2 weeks, 1244 ± 523 ng/mL after 4 weeks, and 1825 ± 489 ng/mL after 10 weeks of 1 mM imatinib exposure. Exposure to 2 mM imatinib resulted in mean serum levels of 6120 ± 1700 ng/mL after 2 weeks, 4856 ± 1882 ng/mL after 4 weeks, and 5843 ± 2977 ng/mL after 10 weeks. In accordance with the half-life of 12.3 hrs of imatinib in rats [19], animals receiving 2 mM imatinib intermittently revealed levels below the detection threshold of the assay (10.0 ng/ml) when serum was collected after four days without drug exposure.

### Long bone length and vertebral height

**Fig 2:**
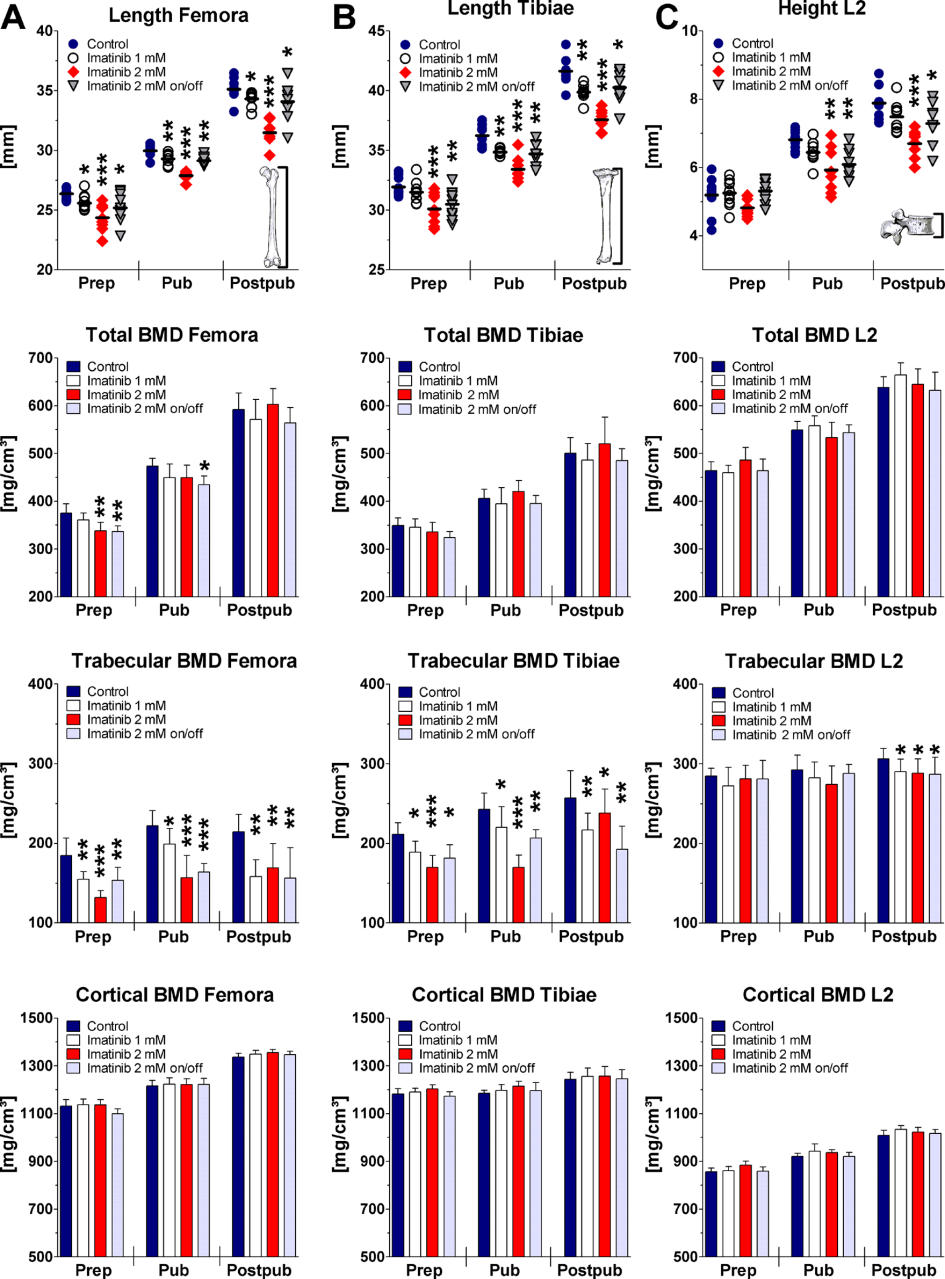
Bone length, total BMD, trabecular BMD, and cortical BMD of the femora (A), tibiae (B), and vertebra L2 (C) during long-term imatinib exposure assessed by pQCT. BMD = bone mineral density; Prep = prepubertal (age of rats: 6 weeks, duration of exposure: 2 weeks; n = 10 each group); Pub = pubertal (age of rats: 8 weeks, duration of exposure: 4 weeks; n = 8 each group); Postpub = postpubertal (age of rats: 14 weeks, duration of exposure: 10 weeks; n = 8 each group); Data represents mean ± 95% CI. Statistical analysis at defined time points: * p<0.05 versus age-related controls; ** p<0.01 versus age-related controls; *** p<0.001 versus age-related controls.

Bone growth in femora and tibiae was found to be reduced under imatinib exposure in a dose-dependent fashion (Fig 2). As analyzed by linear regression, 2 mM imatinib continuous exposure revealed a significant mean bone growth reduction of the femora and tibiae of about 24% and 12%, respectively (p<0.0001). Intermittent exposure to 2 mM imatinib reduced bone growth in femora and tibiae by 8%, which was identical to rats receiving 1 mM imatinib (p = 0.0001; Fig 2). Statistical analysis at all three developmental stages revealed significant bone length reduction of imatinib exposed rats dose-dependently (Fig 2).

Linear regression analysis of the vertebral height revealed non-significantly reduced height of all exposed rats compared to controls during the total 10-week period. However, at defined time points continuous exposure to 2 mM imatinib resulted in a significant height reduction of about 15%, both pubertally and postpubertally (p<0.01). Intermittent exposure to 2 mM imatinib decreased vertebral height significantly by about 10% at pubertal and postpubertal age (p<0.05; Fig 2). Continuous exposure to 1 mM imatinib resulted in a non-significant trend of reduced vertebral height at these time points.

### Bone densities

Linear regression analysis of the long bone BMDs revealed no effect on total BMD of imatinib exposed rats compared to controls during entire exposure time. However, statistical analysis at defined time points revealed that continuous and intermittent exposure to 2 mM imatinib reduced significantly total BMD of the femora by about 10% (p = 0.006) prepubertally, but not at later time points. Total BMD of the tibiae was also reduced prepubertally, but did not reach statistical significance (p = 0.08).

During growth, the physiological increase of trabecular BMD of the femora and tibiae was significantly reduced at all doses applied (p<0.0001). Statistical analysis at defined time points revealed that on average trabecular BMD was significantly reduced by about 30% and 20% in the femora (p<0.01) and tibiae (p<0.01) under continuous exposure to 2 mM imatinib. Under intermittent imatinib exposure trabecular BMD was lowered by about 20% in femora and tibiae at all developmental stages (p<0.01; Fig 2). Trabecular BMD was also significantly lowered in long bones by 15% after exposure to 1 mM imatinib at all developmental stages (p<0.05; Fig 2). However, cortical BMD was not affected by imatinib treatment at all doses applied neither during exposure time nor at defined developmental stages (Fig 2).

Analysis during exposure time of the diaphysis of the long bones revealed that CSA was significantly decreased combined with significantly decreased endostal and periostal circumference of the long bones only by continuous and intermittent exposure to 2 mM imatinib (p<0.001). Statistical analysis at defined time points revealed that CSA was significantly reduced on average by about 10% and 15% in the femora and tibiae under continuous exposure to 2 mM imatinib at all three developmental stages (p<0.05; S1 Table). Endostal and periostal circumference of the femora were significantly decreased on average by 5% (p<0.05) by continuous exposure to 2 mM imatinib at postpubertal stage. At prepubertal and pubertal stage endostal and periostal circumference of the femora were by trend reduced (S1 Table). Endostal and periostal circumference of the tibiae were by trend decreased prepubertally but significantly reduced on average by 10% under continuous exposure to 2 mM imatinib pubertally and postpubertally. Concerning intermittent exposure to 2 mM imatinib, statistical analysis at defined time points revealed by trend reduced femoral and tibial CSA prepubertally, but reduced femoral and tibial CSA on average by 14% at pubertal and postpubertal stage (p<0.05; S1 Table). Endostal and periostal circumferences of the femora were by trend reduced under intermittent exposure to 2 mM imatinib at all developmental stages (p = 0.06). Endostal and periostal circumferences of the tibiae were significantly reduced on average by 8% pubertally and postpubertally (p<0.05; S1 Table). Cortical thickness of the long bones was not affected by imatinib treatment at all doses applied neither during exposure time nor at defined developmental stages during growth.

Statistical analysis of the vertebral BMDs during entire exposure time revealed dose-dependently significantly reduced trabecular BMD under imatinib exposure (p<0.05). Analysis at defined time points disclosed a significant reduction of vertebral trabecular BMD on average by about 5% at all doses applied postpubertally (p<0.05; Fig 2). Vertebral total BMD, cortical BMD, CSA, and cortical thickness were not affected by imatinib treatment (Fig 2; S1 Table).

### Trabecular structure under imatinib exposure

Linear regression analysis of the trabecular structure evaluated by μCT revealed dose-dependently significantly reduced BV/TV and Tb.N (p<0.05) during 10-week period of exposure. Tb.C was also significantly reduced by continuous exposure to 1 mM and 2 mM imatinib during entire exposure time (p<0.05), whereas Tb.Th was not affected. Intermittent treatment to 2 mM imatinib resulted in significantly decreased BV/TV, Tb.Th, Tb.N, (p<0.01), but non-significantly elevated Tb.C during entire exposure time (p = 0.29).

**Fig 3:**
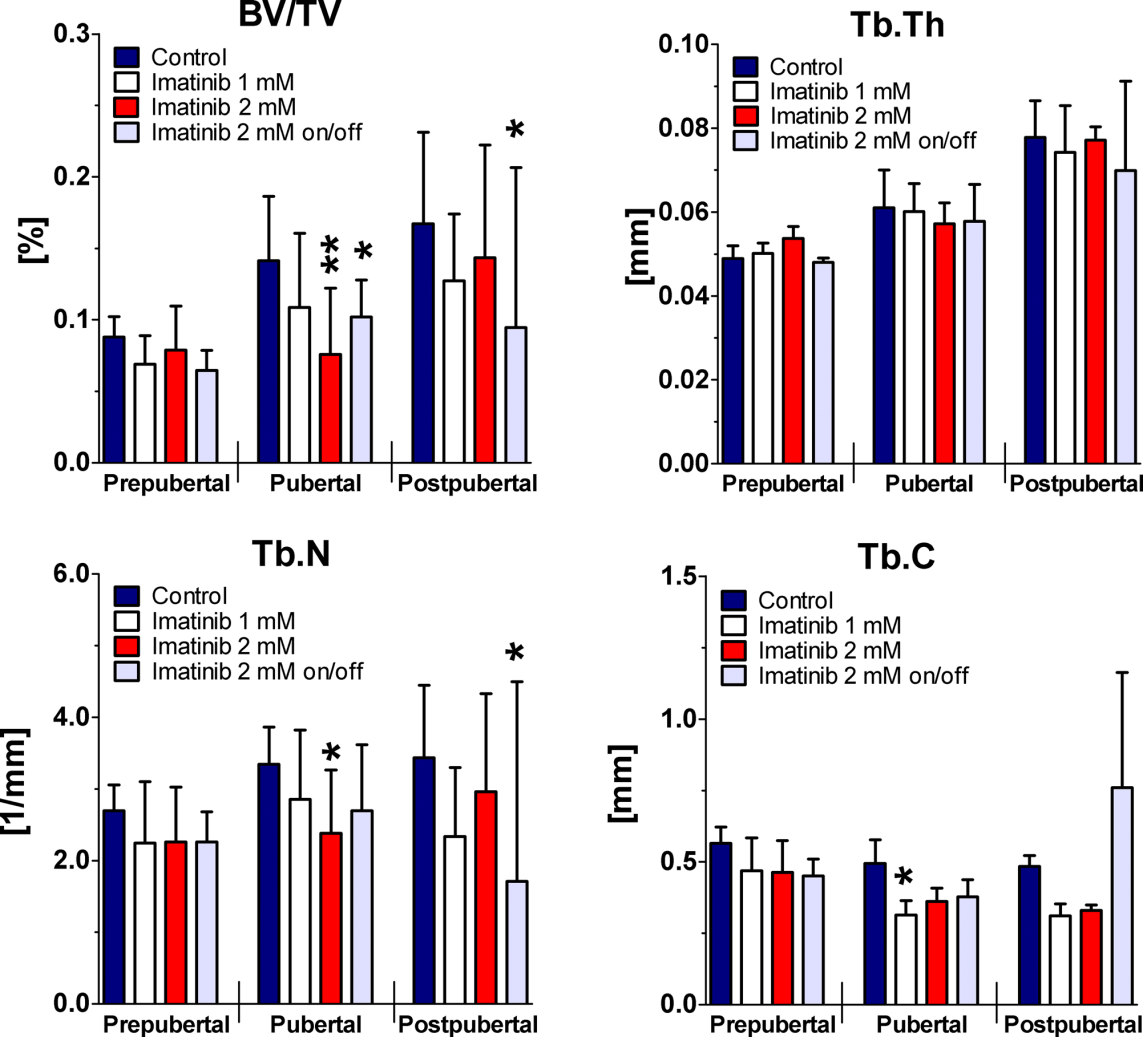
Femoral BV/TV, Tb.Th, Tb.N, and Tb.C during long-term imatinib exposure. Three dimensional trabecular microarchitecture of the right femora was assessed by μCT. Trabecular region of interest was defined manually located within the secondary spongiosa in prepubertal femora 196 slices, pubertal femora 226 slices, and in postpubertal femora 256 slices. For trabecular bone regions, bone volume/ total volume (BV/TV), trabecular thickness (Tb.Th), trabecular number (Tb.N), and trabecular connectivity (Tb.C) were calculated. Data represents mean ± 95% CI. Prepubertal = age of rats: 6 weeks, duration of exposure: 2 weeks (n = 4 each group); Pubertal = age of rats: 8 weeks, duration of exposure: 4 weeks (n = 3 each group); Postpubertal = age of rats: 14 weeks, duration of exposure: 10 weeks (n = 3 each group). Statistical analysis at defined time points: * p<0.05 versus age-related controls; ** p<0.01 versus age-related controls; *** p<0.001 versus age-related controls.

Statistical analysis at defined time points disclosed by trend decreased BV/TV, Tb.N, and Tb.C at all doses applied prepubertally (Fig 3). However, BV/TV was reduced by about 20% under ongoing continuous exposure to 1 mM imatinib pubertally and postpubertally (p = 0.09). This was combined with reduced Tb.N by about 15% pubertally and by about 30% postpubertally (p>0.05). Tb.C was significantly reduced by continuous exposure to 1 mM imatinib pubertally (p = 0.01) but not postpubertally, whereas Tb.Th was not affected at all. Continuous exposure to 2 mM imatinib revealed significantly reduced BV/TV by about 45% pubertally (p = 0.004) and non-significantly postpubertally. This was combined with reduced Tb.N pubertally (p = 0.017) and postpubertally (p>0.05). Tb.C was non-significantly reduced under continuous exposure to 2 mM imatinib at pubertal and postpubertal stage (p = 0.07), whereas Tb.Th was not affected. Intermittent exposure to the high dose resulted in significantly reduced BV/TV by about 25% pubertally (p = 0.04) and by about 40% postpubertally (p = 0.04). This was combined with by trend reduced Tb.N (p = 0.09) pubertally and by about 50% (p = 0.04) postpubertally. Tb.C was found non-significantly reduced combined with unaffected Tb.Th pubertally, but elevated Tb.C (p = 0.34) and reduced Tb.Th (p = 0.09) postpubertally.

### Three-point bending test

**Fig 4:**
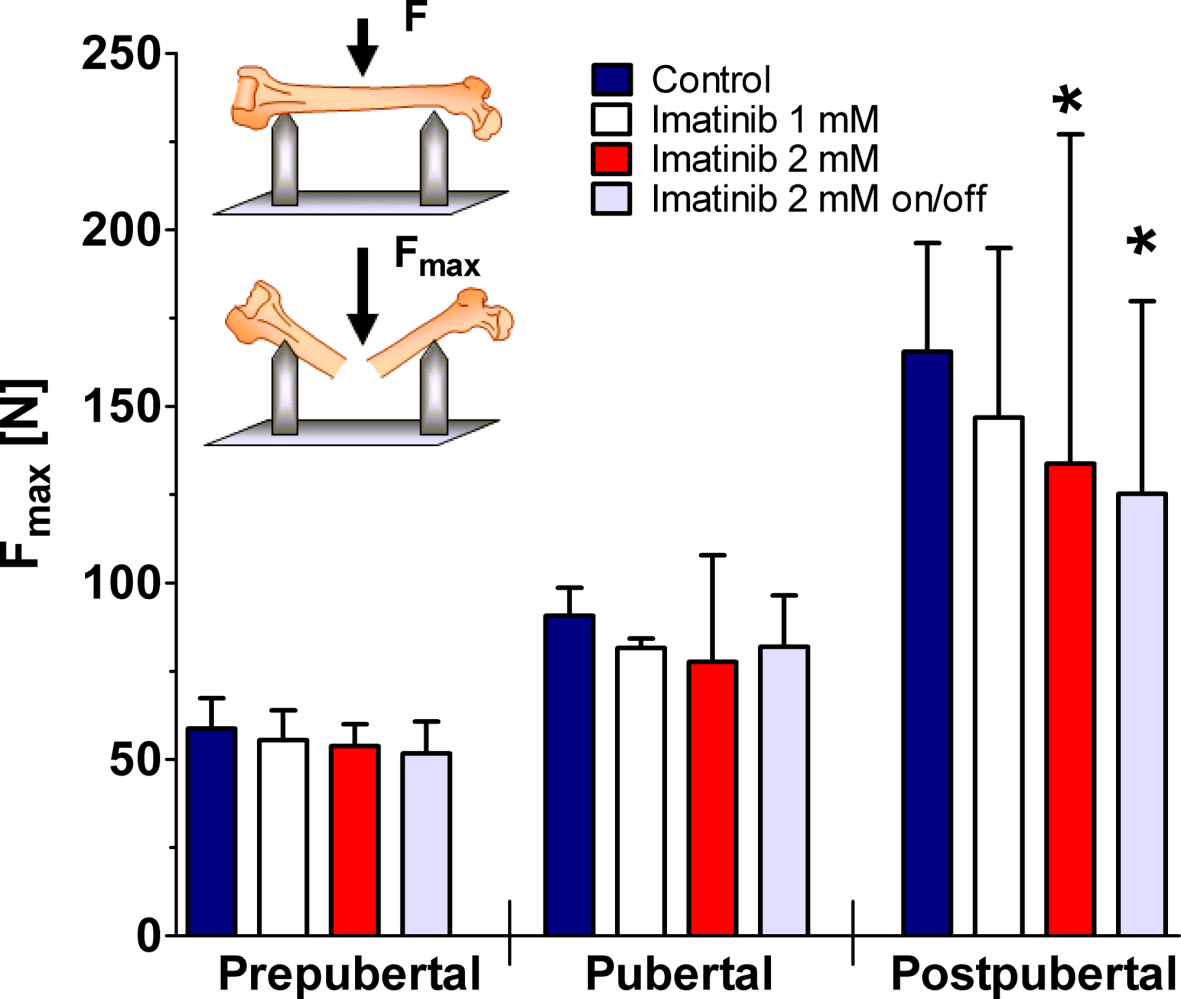
Bending load of the femora during long-term imatinib exposure. Prepubertal = age of rats: 6 weeks, duration of exposure: 2 weeks (n = 4 each group); Pubertal = age of rats: 8 weeks, duration of exposure: 4 weeks (n = 3 each group); Postpubertal = age of rats: 14 weeks, duration of exposure: 10 weeks (n = 3 each group). Data represents mean ± 95% CI. Statistical analysis at defined time points: * p<0.05 versus age-related controls; ** p<0.01 versus age-related controls.

As demonstrated by untreated controls, femoral ultimate tensile strength increased with age. However, continuous and intermittent exposure to 2 mM imatinib caused significantly diminished ultimate tensile strength of the femora during 10-week treatment period (p<0.05; Fig 4). At defined time points, exposure to 1 mM imatinib revealed no effect at all developmental stages investigated. Continuous and intermittent exposure to 2 mM imatinib resulted in a significantly reduced femoral ultimate tensile strength by about 20% in postpubertal rats (p<0.05; Fig 4).

### Biochemical markers of bone turnover

Analysis of serum bone resorption markers during the entire exposure time revealed no significant effect of imatinib treatment on CTX-I levels, whereas TRAP levels were reduced during continuous exposure to 1 mM imatinib (p = 0.07) and 2 mM imatinib (p<0.01). At defined time points, CTX-I levels were non-significantly elevated under continuous exposure to imatinib prepubertally (p = 0.08), but normalized during exposure time. In addition, continuous exposure to imatinib resulted in significantly decreased TRAP levels at all developmental stages. Intermittent exposure to imatinib resulted in by trend decreased TRAP levels at all developmental stages (Table 2). PTH levels during growth revealed dose-dependently significantly elevated levels under continuous imatinib exposure (p<0.01; Table 2). At defined time points, PTH levels were found significantly elevated at all developmental stages under continuous exposure to 2 mM imatinib (Table 2). PTH levels under continuous exposure to 1 mM imatinib were by trend elevated at all developmental stages (p = 0.06; Table 2). Compared to continuous exposure to 2 mM imatinib, intermittent exposure resulted also in elevated PTH levels but not in the same extent (p>0.05; Table 2).

**Table 2:** Altered serum markers of bone turnover during long-term imatinib exposure.

		**Osteocalcin [ng/ml]**	**PINP [ng/ml]**	**CTX-I [ng/ml]**	**TRAP [U/L]**	**PTH [pg/ml]**
**Control**	Prepubertal	861±131	162±55.1	173±73.2	12.3±2.8	16.7±3.5
	Pubertal	790±126	125±73.5	103±20.1	10.0±1.6	11.4±3.7
	Postpubertal	355±33.9	25.8±6.8	31.3±9.1	1.9±0.3	10.1±4.4
**Imatinib 1 mM**	Prepubertal	619±167**	257±175	210±65.1	8.5±1.8*	25.4±6.9
	Pubertal	548±57.3**	147±19.9	82.2±18.3	7.7±1.8	18.8±4.9
	Postpubertal	287±47.0*	33.7±7.1	35.9±5.1	1.3±0.3**	17.6±5.6
**Imatinib 2 mM**	Prepubertal	556±77.7***	198±28.6	219±72.2	5.9±1.0***	32.8±6.9*
	Pubertal	513±94.8**	185±62.9	83.7±33.8	5.8±0.8*	39.9±12.7***
	Postpubertal	303±36.9	41.3±11.4	37.3±6.9	0.8±0.1***	19.2±3.1*
**Imatinib 2 mM on/off**	Prepubertal	948±48.5	149±29.3	154±48.2	11.4±1.9	22.9±12.3
	Pubertal	625±98.5*	172±84.9	62.2±6.0*	7.0±3.7	15.0±4.2
	Postpubertal	319±19.7	46.0±14.7*	49.2±16.9*	1.5±0.2	12.1±5.3

Prepubertal = age of rats: 6 weeks, duration of exposure: 2 weeks (n = 6 each group); Pubertal = age of rats: 8 weeks, duration of exposure: 4 weeks (n = 5 each group); Postpubertal = age of rats: 14 weeks, duration of exposure: 10 weeks (n = 5 each group). Data represents mean ± standard deviation.

Statistical analysis at defined time points:

* p<0.05 versus age-related controls;

** p<0.01 versus age-related controls;

*** p<0.001 versus age-related controls.

Analysis of serum bone formation markers during entire exposure time, disclosed dose-dependently significantly reduced osteocalcin levels under continuous imatinib exposure whereas PINP levels were non-significantly elevated (Table 2). In detail, osteocalcin levels were significantly decreased pre- and pubertally under continuous exposure to imatinib (p<0.01) and normalized postpubertally (Table 2). Intermittent exposure resulted in significantly decreased osteocalcin levels just pubertally (p<0.05). PINP levels under continuous exposure to 1 mM imatinib were by trend elevated during exposure time. Continuous and intermittent exposure to 2 mM imatinib yielded in by trend elevated PINP levels at all developmental stages (Table 2).

## Discussion

Adverse effects stemming from long-term TKI treatment have a higher impact in juvenile individuals with CML as these patients are expected to remain exposed to the drug possibly for their lifetime [20]. So far, skeletal side effects of TKI exposure were analyzed after continuous long-term application in adult rats only or after short-term application of ultra-high doses in newborn rats [11,12,21]. Here we investigated long-term side effects of TKI treatment on the growing skeleton in a growing rat model. TKI exposure as applied in the experiment represents about 10% of the total lifetime of rat covering the prepubertal, pubertal, and postpubertal developmental stages.

The high 97%-bioavailability of imatinib in humans [22] and 62% in rats [23] facilitates oral administration. If dissolved in the drinking water TKI uptake depends on the daily fluid intake, which is age-dependently higher at younger age [24]. Alternate drug administration via oral gavage or intraperitoneal injection—as done in adult rodents—allows daily dosing related to an individual animal’s body weight, but coincides with increased injury risk and stress in juvenile animals [25]. Exposure via drinking water achieved imatinib serum levels at average of 1600 ng/mL during continuous exposure to 1 mM imatinib and at average of 5600 ng/mL during continuous exposure to 2 mM imatinib. This is comparable to therapeutic serum levels in pediatric patients in the range of 2000–8000 ng/mL at imatinib doses of 260–570 mg/m² daily [26] and adult patients in the range of ~1000–3400 ng/mL at imatinib doses of 400–600 mg daily [27,28].

During growth, a 10-week exposure to imatinib causes a significant reduction of the long bone length dose-dependently. In a different approach this was also demonstrated in 5-days old male newborn rats, which received a high dose of imatinib (100–150 mg/kg/d) postnatally for 3 or 9 days only. At the age of 11 weeks, reduced bone length was observed probably due to persisting disorganized growth plate [21]. Our findings match with clinical data in children indicating that continuous administration of imatinib—even in high doses—does not result in a complete stop of growth, rather in a decelerated growth rate of the long bones [7–10,14,29–31].

pQCT analysis of the bones during growth revealed significantly reduced trabecular BMD by imatinib exposure. Analysis of the 3D trabecular structure by μCT emphasize this result by reduced BV/TV combined with reduced Tb.N and Tb.C. Comparable to adult rats, 5-week imatinib treatment at doses of 40 or 71 mg/kg/d, resulted in reduced trabecular BMD [11]. These results contrast clinical observations in older adult patients showing increased BMD and bone mass under imatinib therapy [6,32,33]. However, our findings also indicate unchanged cortical BMD and cortical thickness during growth dose- and time-independently, whereas bone strength of the femora was decreased after long-term exposure to high dose imatinib. This could be explained by observed decreased CSA, and periostal and endostal circumference of the femora (S1 Table) suggesting a blunted radial appositional bone growth. With regard to pediatric patients BMD measurements or increased fracture rates under long-term imatinib treatment are not published yet.

As new perspective, we could demonstrate that intermittent imatinib exposure will ameliorate growth impairment in rats. This approach might reduce some skeletal side effects in pediatric patients [14]. A single trial in older adults has already proven that intermittent TKI treatment is sufficient to control CML once remission has been achieved [13]. However, this approach does not recover biomechanical strength of the long bones in rats.

Imatinib exposure significantly reduced vertebral height combined with decreased trabecular BMD [34]. Up to now, only limited and contrary data are available on this issue. As assessed by DXA in adult patients with CML, O´Sullivan *et al*. observed significantly increased lumbar spine BMD after 24 month of imatinib treatment [35], whereas Vandyke *et al*. observed unchanged BMD [6]. Data from pediatric patients or juvenile animal models are not published yet. We conclude that imatinib also alters vertebral properties, but not to the same extend as in long bones.

The bone resorption marker TRAP revealed significant decreased serum levels under continuous imatinib exposure indicating reduced osteoclast number at all developmental stages. This is confirmed by *in vitro* studies showing that imatinib impairs osteoclastogenesis leading to diminish numbers of TRAP-positive osteoclasts [36–41]. However, when the bone resorption marker CTX-I was investigated in parallel, serum levels were by trend elevated prepubertally, but normalized during ongoing exposure time, indicating nearly unchanged osteoclast activity during growth. This finding is consistent with data from pediatric patients with CML describing by trend elevated CTX-I levels prepubertally while on imatinib [42].

Furthermore, adult patients with CML on imatinib treatment frequently develop secondary hyperparathyroidism [5,35,43,44]. Also 50% of pediatric CML patients display hyperparathyroidism independently of duration of imatinib therapy [42]. The juvenile rat model also unraveled hyperparathyroidism during the entire drug exposure time. However, intermittent exposure to imatinib revealed levels of PTH and TRAP comparable to controls indicating a compensatory mechanism during TKI exposure breaks.

Investigation of serum bone formation markers showed decreased osteocalcin but by trend elevated PINP levels, pointing to improved bone formation and mineralization under imatinib exposure. *In vitro* assays using human isolated mesenchymal stem cells, primary rat osteoblasts, and mouse osteoblast-like cell line MC3T3-E1 revealed all increased mineralization combined with reduced proliferation under therapeutic imatinib concentration [45]. However, clinical data are not consistent: in adult patients with CML reduced osteocalcin levels [5] as well as elevated osteocalcin and PINP levels have been reported after 3 months of imatinib treatment. However, normalization occurring after 18 months of TKI therapy indicated a biphasic response of bone formation [35,46]. Pediatric patients with CML exhibited initially elevated osteocalcin levels followed by a significant decline of 0.3 μg/L per week on imatinib treatment [42].

The detailed mechanism how imatinib impairs bone remodeling and growth is speculative yet. Our data match to the report by Nurmio *et al*. describing negatively altered bone remodeling at the metaphyseal osteochondral junction after single high dose administration postnatally [21]. Data of Nurmio *et al*., Vandyke *et al*.[12], and our data, uniformly support the hypothesis that imatinib exposure alters metabolism and remodeling of the growing bone in a temporal-spatial stepwise fashion. In the first instance, migration, proliferation, and activity of chrondrocytes will be impaired by imatinib [12,21] leading to a disturbed organization of the growth plate impairing longitudinal bone growth [21]. Disturbed growth hormone secretion under imatinib treatment as shown before in not-outgrown organism [7,8,47] may aggravate growth impairment. Thereafter, ongoing drug exposure causes a spatial activity shifting of bone remodeling: initially formation will be elevated and shifted to the area of the osteochrondral junction, whereas activity of bone resorption remains unchanged but will be spatially shifted to the distal area of the trabecular bone [21]. Finally, under long-term imatinib treatment osteoblastogenesis and osteoclastogenesis will be impaired [40,48], hampering bone remodeling during growth. This multistep hypothesis is supported by clinical observations in adult and pediatric patients with CML showing a biphasic response of bone formation with increasing bone formation at the beginning and declining bone formation with ongoing imatinib therapy [35,42].

## Conclusion

The juvenile rat model represents an appropriate model to examine the side effects of long-term TKI exposure on the growing bone in a developmental stage-dependent fashion. Impairment of longitudinal growth as observed in children under imatinib treatment could be unequivocally modeled and confirmed. The hypothesis of spatio-temporal shifting of skeletal formation and resorption is in line with the wide spectrum of inhibition exerted by TKI on osteoclasts and osteoblasts, thus impairing bone remodeling at various levels. Furthermore, our study indicates that long-term imatinib exposure may result in reduced bone strength possibly posing an elevated fracture risk on pediatric patients. Intermittent imatinib treatment may reduce skeletal effects on the growing bone and, therefore, could improve the risk-benefit ratio of long-term TKI exposure in pediatric patients.

## Supporting Information

S1 Fig Body weight of the rats during long-term imatinib exposure. Body weight was measured 3 times weekly (Monday, Wednesday, Friday). Data represents mean ± 95% CI.
(TIF)

S1 Table Skeletal parameters of the femora, tibiae, and vertebrae (L2) during long-term imatinib exposure measured by pQCT. CSA = cross-sectional area; C.Th = cortical thickness; Prepubertal = age of rats: 6 weeks, duration of exposure: 2 weeks; n = 10 each group; Pubertal = age of rats: 8 weeks, duration of exposure: 4 weeks; n = 8 each group; Postpubertal = age of rats: 14 weeks, duration of exposure: 10 weeks; n = 8 each group. Data represents mean ± standard deviation. Statistical analysis at defined time points: a: p<0.05 versus age-related controls; b: p<0.01 versus age-related controls; c: p<0.001 versus age-related controls.
(DOCX)
