# Supplementary figures and images for: Impact of Long-Term Exposure to the Tyrosine Kinase Inhibitor Imatinib on the Skeleton of Growing Rats
Source: PLoS One. 2015 Jun 24;10(6):e0131192. doi: 10.1371/journal.pone.0131192 (PMC4479438; doi:10.1371/journal.pone.0131192)

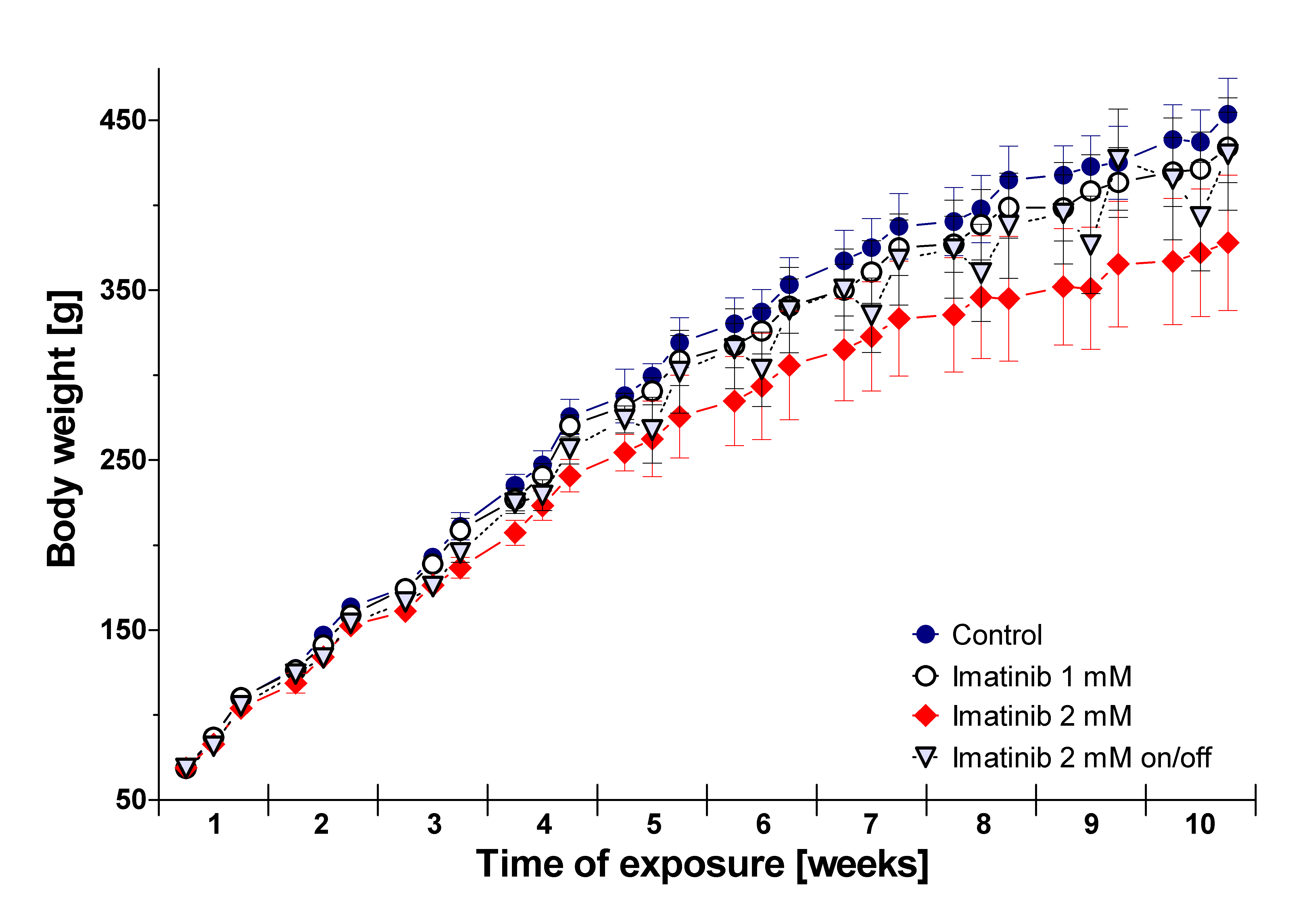

Supplement: S1 Fig — Body weight was measured 3 times weekly (Monday, Wednesday, Friday). Data represents mean ± 95% CI. (TIF) [file pone.0131192.s001.tif]
