# Supplementary material for: Impact of Long-Term Exposure to the Tyrosine Kinase Inhibitor Imatinib on the Skeleton of Growing Rats
Source: PLoS One. 2015 Jun 24;10(6):e0131192. doi: 10.1371/journal.pone.0131192 (PMC4479438; doi:10.1371/journal.pone.0131192)
Supplement: S1 Table — CSA = cross-sectional area; C.Th = cortical thickness; Prepubertal = age of rats: 6 weeks, duration of exposure: 2 weeks; n = 10 each group; Pubertal = age of rats: 8 weeks, duration of exposure: 4 weeks; n = 8 each group; Postpubertal = age of rats: 14 weeks, duration of exposure: 10 weeks; n = 8 each group. Data represents mean ± standard deviation. Statistical analysis at defined time points: a: p<0.05 versus age-related controls; b: p<0.01 versus age-related controls; c: p<0.001 versus age-related controls. (DOCX) [file pone.0131192.s002.docx]

**Supporting Information**

**S1 Table. Skeletal parameters of the femora, tibiae, and vertebrae (L2) during long-term imatinib exposure measured by pQCT.**

|  |  | **Femora** | | | | **Tibiae** | | | | **Vertebrae** | |
| --- | --- | --- | --- | --- | --- | --- | --- | --- | --- | --- | --- |
|  |  | **CSA (mm²)** | **C.Th (mm)** | **Circumference (mm)** | | **CSA (mm²)** | **C.Th (mm)** | **Circumference (mm)** | |  |  |
|  |  |  |  | **Endost** | **Periost** |  |  | **Endost** | **Periost** | **CSA (mm²)** | **C. Th (mm)** |
| **Control** | Prepubertal | 8.76±0.65 | 0.37±0.02 | 8.15±0.46 | 10.5±0.39 | 4.74±0.32 | 0.36±0.02 | 5.48±0.33 | 7.72±0.26 | 5.81±0.70 | 0.13±0.03 |
|  | Pubertal | 10.4±0.32 | 0.53±0.04 | 7.85±0.31 | 11.2±0.32 | 6.86±0.51 | 0.44±0.02 | 6.49±0.39 | 9.28±0.35 | 6.69±0.93 | 0.22±0.02 |
|  | Postpubertal | 12.5±1.06 | 0.68±0.02 | 8.28±0.63 | 12.5±0.52 | 12.0±1.81 | 0.57±0.03 | 8.35±1.16 | 11.9±1.01 | 7.43±0.90 | 0.33±0.03 |
| **Imatinib 1 mM** | Prepubertal | 8.67±0.62 | 0.38±0.02 | 8.06±0.47 | 10.4±0.37 | 4.72±0.37 | 0.37±0.02 | 5.38±0.36 | 7.69±0.31 | 5.55±0.63 | 0.13±0.02 |
|  | Pubertal | 10.3±0.76 | 0.53±0.02 | 8.04±0.41 | 11.4±0.43 | 6.44±0.46 | 0.45±0.01 | 6.16±0.27 | 8.99±0.32 | 6.01±0.66 | 0.23±0.02 |
|  | Postpubertal | 12.1±0.86 | 0.69±0.03 | 7.95±0.45 | 12.3±0.44 | 10.2±0.86 | 0.62±0.03 | 7.36±0.40 | 11.3±0.48 | 7.33±0.79 | 0.37±0.04 |
| **Imatinib 2 mM** | Prepubertal | 7.98±0.64^a^ | 0.36±0.03 | 7.74±0.43 | 10.1±0.41^a^ | 4.41±0.39 | 0.36±0.03 | 5.16±0.30 | 7.44±0.32 | 5.14±0.39 | 0.15±0.03 |
|  | Pubertal | 9.46±0.57^b^ | 0.52±0.04 | 7.65±0.39 | 10.9±0.33 | 5.72±0.44^c^ | 0.47±0.03 | 5.54±0.40^b^ | 8.48±0.33^b^ | 5.86±0.48 | 0.21±0.04 |
|  | Postpubertal | 11.2±0.56^a^ | 0.69±0.03 | 7.55±0.24^a^ | 11.9±0.29^a^ | 8.40±0.89^b^ | 0.64±0.05 | 6.21±0.47^b^ | 10.3±0.55^b^ | 6.15±0.40 | 0.32±0.04 |
| **Imatinib 2 mM on/off** | Prepubertal | 8.37±0.67 | 0.37±0.02 | 7.92±0.48 | 10.3±0.41 | 4.66±0.36 | 0.35±0.02 | 5.46±0.26 | 7.65±0.29 | 5.80±0.49 | 0.13±0.02 |
|  | Pubertal | 9.15±0.31^b^ | 0.50±0.02 | 7.57±0.19 | 10.7±0.18 | 5.87±0.34^c^ | 0.43±0.02 | 5.91±0.32^b^ | 8.58±0.24^b^ | 5.98±0.46 | 0.21±0.02 |
|  | Postpubertal | 10.0±0.79 | 0.67±0.05 | 7.90±0.62 | 12.1±0.83 | 10.2±0.45^a^ | 0.59±0.06 | 7.50±0.95^a^ | 11.2±1.18 | 7.24±1.13 | 0.32±0.05 |

CSA= cross-sectional area; C.Th =cortical thickness; Prepubertal=age of rats: 6 weeks, duration of exposure: 2 weeks; n=10 each group; Pubertal=age of rats: 8 weeks, duration of exposure: 4 weeks; n=8 each group; Postpubertal=age of rats: 14 weeks, duration of exposure: 10 weeks; n=8 each group. Data represents mean ± standard deviation.

Statistical analysis at defined time points: a: p<0.05 versus age-related controls; b: p<0.01 versus age-related controls; c: p<0.001 versus age-related controls
